# TSEE: an elastic embedding method to visualize the dynamic gene expression patterns of time series single-cell RNA sequencing data

**DOI:** 10.1186/s12864-019-5477-8

**Published:** 2019-04-04

**Authors:** Shaokun An, Liang Ma, Lin Wan

**Affiliations:** 1 Academy of Mathematics and Systems Science, Chinese Academy of Sciences, Beijing, 100190 China; 2 Beijing Institute of Genomics, Chinese Academy of Sciences, Beijing, 100101 China; 3 University of Chinese Academy of Sciences, Beijing, 100049 China

**Keywords:** Nonlinear dimensionality reduction, Elastic embedding, Visualization, Single-cell RNA sequencing, Time series, Cell fate decisions, Gene expression pattern, Oscillation, In-group proportion

## Abstract

**Background:**

Time series single-cell RNA sequencing (scRNA-seq) data are emerging. However, the analysis of time series scRNA-seq data could be compromised by 1) distortion created by assorted sources of data collection and generation across time samples and 2) inheritance of cell-to-cell variations by stochastic dynamic patterns of gene expression. This calls for the development of an algorithm able to visualize time series scRNA-seq data in order to reveal latent structures and uncover dynamic transition processes.

**Results:**

In this study, we propose an algorithm, termed time series elastic embedding (TSEE), by incorporating experimental temporal information into the elastic embedding (EE) method, in order to visualize time series scRNA-seq data. TSEE extends the EE algorithm by penalizing the proximal placement of latent points that correspond to data points otherwise separated by experimental time intervals. TSEE is herein used to visualize time series scRNA-seq datasets of embryonic developmental processed in human and zebrafish. We demonstrate that TSEE outperforms existing methods (e.g. PCA, tSNE and EE) in preserving local and global structures as well as enhancing the temporal resolution of samples. Meanwhile, TSEE reveals the dynamic oscillation patterns of gene expression waves during zebrafish embryogenesis.

**Conclusions:**

TSEE can efficiently visualize time series scRNA-seq data by diluting the distortions of assorted sources of data variation across time stages and achieve the temporal resolution enhancement by preserving temporal order and structure. TSEE uncovers the subtle dynamic structures of gene expression patterns, facilitating further downstream dynamic modeling and analysis of gene expression processes. The computational framework of TSEE is generalizable by allowing the incorporation of other sources of information.

## Background

Single-cell RNA sequencing (scRNA-seq) technology provides snapshots of transcriptomes at single-cell resolution, offering a comprehensive approach to study complex biological processes, such as cell fate decisions [1–3]. Given the challenges raised by high-dimensional gene expression profiles of large-scale heterogeneous cell populations at diverse stages during cell state-transitions, many computational methods have been proposed for the visualization, clustering, and reconstruction of scRNA-seq data (see [4], a recent review).

Cells used for a single-cell experiment are snapshot of heterogeneous dynamic populations. However, the dynamic range is usually limited, and only specific cell fate decisions can be analyzed when the samples are collected at a single stage or condition. Therefore, to extend the dynamic range and account for whole processes of cell development, time series scRNA-seq data are emerging. These time series data are generated by sampling single cells collected and sequenced at multiple time stages along the time course of cell developmental processes [5, 6]. Yet, by integrating time series analysis into scRNA-seq, new questions and challenges arise. Importantly, we know that scRNA-seq data across time stages are contaminated by assorted sources of variations during data collection and generation [7]. Moreover, the cell-to-cell gene expression is highly variable across the stochastic dynamics of gene transcription [8]. To address these issues, a handful computational time series scRNA-seq methods have been developed to (1) determine temporal trajectories [9], (2) reconstruct cell development landscapes based on the sophisticated mathematical tool of optimal transport [10], and (3) identify gene-gene interactions, as well as gene networks [8, 11].

However, for such methods to perform model reduction, computation and validation, they must incorporate dimensionality reduction and/or visualization of single-cell high-dimensional gene expression profiles. Accordingly, dimensionality reduction would ideally reveal the intrinsic structure of the data by representing both global structure by preserving the topology and geometry and local structure by preserving the neighborhood relationship. Among the numerous dimensionality reduction and visualization methods, principal component analysis (PCA) and t-distributed stochastic neighbor embedding (tSNE) are most widely used in the single-cell community to visualize data structures [4, 12]. However, PCA is designed to preserve linear structure based on eigen-decomposition of a matrix into canonical form, and as such, it is incapable of handling nonlinear structures. While tSNE emphasizes neighborhood information to reveal the local cluster structures of the data, it tends to shatter trajectories and fails to preserve the global structures [12].

To address the limits of dimensionality reduction methods, like PCA and tSNE, we recently adopted the nonlinear dimensionality reduction algorithm, Elastic Embedding (EE) [13], to accurately visualize and reconstruct the embedded intrinsic latent space of cell development trajectory[14]. Both tSNE and EE embed high-dimensional data points into low-dimensional latent space by modeling the data points interactively with two terms. One term is an attractive force that attracts pairs of points towards each other, while the other term activates a repulsive force that simultaneously separates all pairs of points. A similar embedding idea was adopted in force-directed layout embedding [15], which has also been used in the visualization of scRNA-seq [16]. As an extension of tSNE, the EE algorithm penalizes placing latent points far from similar data points, as well as penalizes placing latent points from dissimilar data points close together [13], thereby preserving both local and global intrinsic data structures [17].

However, to the best of our knowledge, no dimensionality reduction and visualization methods can now incorporate temporal information of single-cell experiments into times series scRNA-seq data, i.e., time stages when samples are collected. Instead, scRNA-seq data from different time stages are combined as the input for methods like PCA and tSNE to obtain 2-dimensional visualization of the data structure [5, 6, 10]. TSEE can carefully incorporate experimental temporal information, resulting in significant improvement of temporal resolution of the cells on the 2-dimensional plane, as well as uncovering the subtle structures of dynamic gene expression patterns.

In this study, we propose a time series elastic embedding (TSEE) algorithm for dimensionality reduction and visualization of time series scRNA-seq data by incorporating the temporal information of the experiments. TSEE is an extension of EE by introducing an additional repulsive force term when pairs of data points are collected at distinct time stages (see Eq. 1). TSEE penalizes placing latent points in close proximity to data points otherwise separated by experimental time interval. For developmental processes, the rationale that underlies TSEE holds that data points sampled at the same time stage should be more similar than those sampled from adjacent time stages, while those data points sampled from adjacent time stages should be more similar than data points sampled from the nonadjacent time stages. In this way, TSEE preserves the temporal order and structure of time series data through the input of experimental temporal information. A similar motivation was successfully applied in the time-dependent community detection of time-varying networks [18].

In this paper, we first introduce the TSEE algorithm, and then provide an efficient numerical implementation of TSEE based on the partial-Hessian method developed by EE [19]. Next, we demonstrate the power of TSEE in the visualization of two datasets of time series scRNA-seq: human preimplantation embryo (hereinafter denoted as HPE) dataset [5] and early zebrafish embryonic development (hereinafter denoted as Zebrafish) dataset [6]. By establishing a new constraint term of temporal repulsive force, TSEE dilutes the distortions of the assorted sources of data variations across time stages and achieves temporal resolution enhancement. Compared to existing methods such as PCA, tSNE and EE, our TSEE shows superior ability to gain time resolution on 2-dimensional space by preserving local, global and temporal structures of time series scRNA-seq data. Furthermore, the visualization represented by TSEE uncovers the subtle patterns of dynamic gene expression by showing continuous patterns with regularity at the interface between samples from adjacent time stages. For example, TSEE reveals the oscillating waves of gene expression for *HER1*, *HER7*, *SOX2* and etc. along the time course, providing a solid foundation for downstream mathematical modeling and analysis. We also demonstrate robustness in the choice of TSEE.

## Methods

### Time series elastic embedding (TSEE)

Given the time series scRNA-seq dataset, the single-cell samples are collected at *n* time stages {*t*_1_,*t*_2_,…,*t*_*n*_}, and for each time stage *t*_*i*_(1≤*i*≤*n*), a number of *m*_*i*_ single cells are sequenced with the corresponding gene expression vectors $y_{1}^{(t_{i})},\ldots,y_{m_{i}}^{(t_{i})} \in \mathbb {R}^{D}$, where *D* is the number of genes for single cells. Thus, the single cell gene expression profile of a total number of $N=\sum _{i=1}^{n} m_{i}$ cells is contained in the data matrix

$$Y_{N \times D} = \left(y_{1}^{(t_{1})}, \ldots, y_{m_{1}}^{(t_{1})}, \ldots, \ldots, y_{1}^{(t_{n})} \cdots, y_{m_{n}}^{(t_{n})} \right)^{T}. $$

TSEE is an extension of EE, a nonlinear dimensionality reduction method [13] by incorporating the information of *t*_*i*_, embedding the *N* data points in *D*-dimensional space into the latent *d*-dimensional (*d*≪*D*) coordinates of

$$X_{N\times d} = \left(x_{1}^{(t_{1})}, \ldots, x_{m_{1}}^{(t_{1})}, \ldots, \ldots, x_{1}^{(t_{n})} \cdots, x_{m_{n}}^{(t_{n})} \right)^{T}\, {.} $$

In a manner that minimizes the pseudo potential energy function as

1 $$ {\begin{aligned} E \left[ X;\lambda,\beta \right] \,=\, \!\!\sum\limits_{n,m=1}^{N} w_{nm}^{P} \|x_{n}\,-\,x_{m}\|^{2} \,+\,\lambda \sum\limits_{n,m=1}^{N} \left(w_{nm}^{N} \,+\, \beta t_{nm} \right) \exp \left(\,-\,\|x_{n}\,-\,x_{m}\|^{2} \right), \end{aligned}}  $$

where the weights $w_{nm}^{P},w_{nm}^{N},t_{nm} $ are defined as

$$\begin{array}{*{20}l} w_{nm}^{P} &= N^{+} w_{nm}^{+} = N^{+}\exp\left(\frac{-\|y_{n}-y_{m}\|^{2}}{2\sigma^{2}}\right),\\ w_{nm}^{N}&= N^{-} w_{nm}^{-}=N^{-}\|y_{n}-y_{m}\|,\\ t_{nm}&=N^{-}|t(n)-t(m)|. \end{array} $$

Among these, *N*^+^ and *N*^−^ are normalization factors of weights for the two summation terms which, respectively, are $N^{+} = \left (\sum _{n,m=1}^{N} {w_{nm}^{+}} \right)^{-1}$ and $N^{-} = \left (\sum _{n,m=1}^{N}{w_{nm}^{-}} + \beta |t(n)-t(m)| \right)^{-1}$; |*t*(*n*)−*t*(*m*)| is the time interval between sample *y*_*n*_ and *y*_*m*_. TSEE is different from EE by incorporating additional temporal information of *t*_*nm*_ in the model.

The pseudo potential energy function of TSEE has two terms on the right hand side of Eq. 1. The first term (*attractive* term) attracts pairs of data points towards each other, while the second term (*repulsive* term) separates all pairs of points. The weights $W^{P}=\left (w_{nm}^{P} \right)$ of the *attractive* term are defined based on the similarity between data points in high-dimensional space (original space) [20], enabling TSEE to place neighboring points in high-dimensional space still close to each other in low-dimensional embedding space. The weights of the *repulsive* term of TESS are composed of two parts. The weights $W^{N}=\left (w_{nm}^{N}\right)$ are the disparities between data points in high-dimensional space (original space), and *T*^−^=(*t*_*nm*_) are the time intervals between samples. Thus, in the constructed latent space, TSEE not only preserves the local and global structures of data hidden in original high-dimensional space, as EE does [14, 17], but also further preserves temporal structure of data by penalizing placing close together latent points that correspond to data points separated by experimental time intervals. Intuitively, the experimental temporal information of the experimental time of samples reflects, to some extent, cell development stages. In other words, the later a cell is collected in time, the more likely it will be in later developmental stages, and a longer experimental time interval between two cells typically indicates a longer distance between the cells, along the developmental process.

TSEE has two regularization parameters in Eq. 1. Parameter *λ* trades off the attractive and repulsive terms, while parameter *β* trades off the composite weights of data disparities in high-dimensional space and time intervals on the repulsive term. By fixing *λ*, a smaller *β* will have smaller effects by the temporal order/structure of samples, leading to less consideration of temporal information. If *β*=0, TSEE degenerates into EE, and no temporal information will be incorporated. If fixing *β*, a smaller *λ* makes TSEE focus on local structures, determining *X*_*N*×*d*_ based on the attractive term, and when *λ*=0, TSEE degenerates to Laplacian Eigenmap methods [13]. Choices and robustness analysis of both *λ* and *β* on embeddings in latent space are discussed in “Results” section. If either *λ* or *β* is too large, the result will either be distortion of local structures or loss of global structures in latent space.

The pseudocode of TSEE is in Algorithm 1.


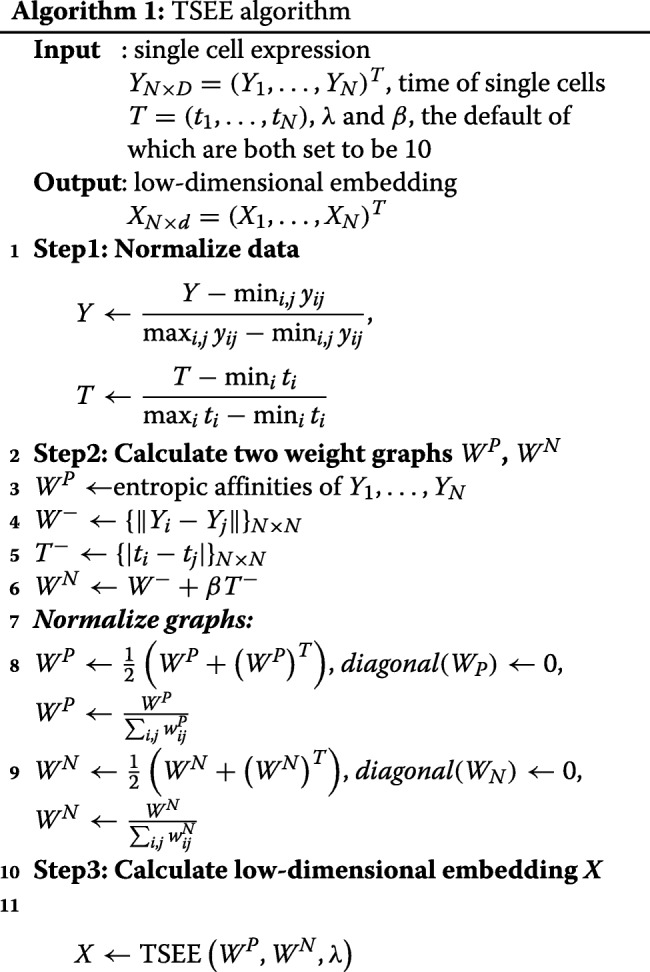


#### The numerical implementation of TSEE

In the numerical implementation of TSEE, we adopt the efficient computational method of partial-Hessian proposed in [19] to find *X*_*N*×*d*_ that minimizes the function *E* of TSEE (Eq. 1).

For a given symmetric weighted graph (matrix) *W*=(*w*_*nm*_), the Laplacian matrix graph is defined as *L*=*D*−*W*, where $D = \text {diagonal}\left (\sum _{m=1}^{N} w_{nm}\right)$ is defined as degree matrix. *L* is positive semi-definite if *W* is non-negative. The gradient of objective function *E* of TSEE can be represented in terms of Laplacian as

$$\nabla E = 4 LX, $$

where *L* is the Laplacian matrix of *W*=(*w*_*nm*_) with

$$w_{nm} = w_{nm}^{P} -\lambda \left(w_{nm}^{N} + \beta t_{nm}\right)\exp\left(-||x_{n}-x_{m}||^{2}\right). $$

This optimization problem can be solved by an iterative approach in the form of

$$x_{k+1} = x_{k}+\alpha_{k}p_{k}, $$

where *α*_*k*_>0 is the step size in each iteration that satisfies the linear search principles (e.g., Armijo rule), and $p_{k} \in \mathbb {R}^{ND}$ is the search direction. In each iteration, *p*_*k*_ is determined by

$$B_{k}p_{k}=-g_{k}, $$

where *g*_*k*_ is the gradient at *k*-th iteration, and *B*_*k*_ is a positive-definite matrix to achieve a descent direction satisfying $p_{k}^{\mathrm {T}}g_{k}<0$. The procedures iterate until certain conditions are satisfied, for example, the iteration terminates when the number of iterations achieves the given constraint, or the distance between *x*_*k*_ and *x*_*k*+1_ obtained by two adjacent iterations is less than a given threshold.

The partial-Hessian method relies on the *spectral direction* based on partial Hessian, the attractive Hessian *L*^*P*^⊗*I*_*d*_, to strike the best compromise between deep descent and efficient computation, where *L*^*P*^ is the Laplacian matrix of *W*^*P*^. In order to prevent the problem of singularity, a small *μ*_*k*_*I* is added to the partial Hessian, ensuring positive definiteness of *B*_*k*_, that is

$$B_{k} = L^{P} \otimes I_{d} + \mu_{k} I $$

and *μ*_*k*_ is set as 10^−10^ min*i*,*j*(*L*^*P*^)_*i*,*j*_ in this study. Algorithm 2 shows the detailed steps of Partial-Hessian strategies. The authors of EE [19] demonstrated that the spectral direction obtained, as described above, can be rapidly computed, leading to global and fast convergence. Compared with EE, TSEE adds an additional repulsive factor in the objective function, which results in a simple modification of *W* and *L* with no effect on computational performance, making the complexity of TSEE comparable to that of EE. In terms of the large-scale Zebrafish dataset with sample size ∼40*k*, the computational time of EE and TSEE is 38 min and 45 min, respectively.


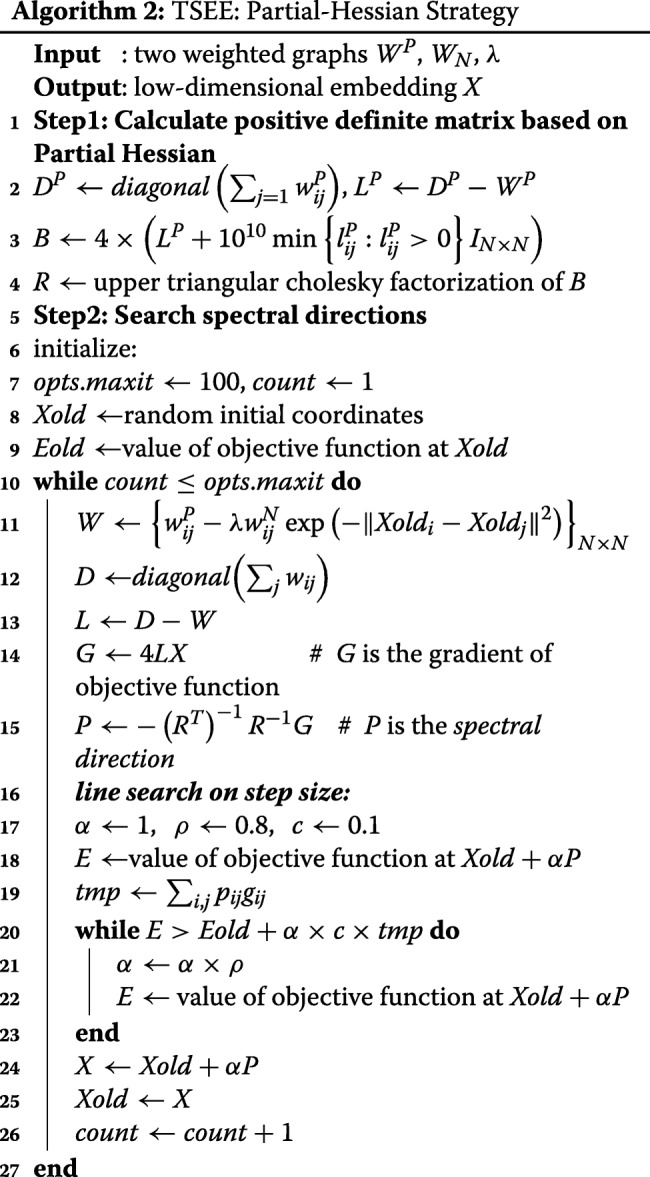


#### Datasets

Two publicly available datasets of time series scRNA-seq are used in this study. The Zebrafish dataset [6] was obtained across 12 closely spaced stages of early zebrafish development spanning from high blastula stage (3.3 hours-postfertilization (hpf), just after transcription from the zygotic genome begins) to six-somite stage (12 hpf, shortly after the completion of gastrulation). The number of cells at each time stage ranges from 200 to 7162, comprising a total of 39,505 cells. The UMI count data can be accessed at NCBI GEO (accession no.GSE106587). The human preimplantation embryo (HPE) dataset [5] consists of individually isolated embryonic cells during the human preimplantation process, starting from the 8-cell stage at embryonic day 3 (E3) up to the time point just prior to implantation at E7, consisting of a total of 1529 single cells. The data can be downloaded from https://www.ebi.ac.uk/arrayexpress/experiments/E-MTAB-3929/.

In addition, the scRNA-seq dataset of *Drosophila* embryos [21] is utilized here as a demonstration of the generalizability of TSEE for incorporating spatial information. The high-quality data of a total 1297 cells, as defined in [21], were selected. The data are used to reconstruct the spatial positions of cells in the precisely staged embryos. The 84 genes used as predictors of spatial positions of cells in the precisely staged embryos as in [21] are utilized as our spatial genes. The data can be obtained at https://shiny.mdc-berlin.de/DVEX/.

#### Preprocessing of time series scRNA-seq data

Prior to the implementation of TSEE, the time series scRNA-seq data are preprocessed as follows. First, we select out the most variable genes according to the *Z*-scores of their variations across all samples. Second, the selected gene expression profile is normalized with all values, subtracting the minimum in the matrix, followed by dividing all values by their maximum of the processed matrix, such that the values of the elements of the normalized profile matrix range from 0 to 1. The time points of samples are also normalized to range from 0 to 1 with the initial time stage set as 0 and the final time stage set as 1. Third, the PCA algorithm is utilized to select the top principal components which preserve most variations, and the dimension of components is determined following the procedure in [14]. Finally, the data of top PCA components are further normalized, as described in the second step.

#### Performance comparisons

We compare TSEE with PCA, tSNE and EE to evaluate their individual performance on visualizing data, preserving local and global structures, and revealing gene expression patterns.

The scatting plots of cells on the 2-dimensional embedded space are first displayed to compare their visualization results. A good visualization result should reveal the local and global structures, as well as uncover the latent specific gene expression patterns.

To measure the preservation of structures quantitatively, we adopt 1) the in-group proportion (*IGP*) [22] and 2) *IGP2*, a modification of *IGP*, to evaluate local structure preservation. In addition, we adopt the Pearson correlation coefficient (PCC) between experimental time and pseudotime obtained by DensityPath [14], to evaluate global structure preservation.

The *IGP*, defined as the proportion of samples in a group, the nearest neighbors of which are also in the same group [22], can be used to assess the accuracy of distinction of cells on 2-dimensional space. In this study, we use temporal information to define groups. For a given dataset *X*={*x*_1_,*x*_2_,…,*x*_*N*_}, consisting of *N* samples which belong to *n* groups *U*={1,2,…,*u*,…,*n*}, we define *j*^*N*^= arg min*k*≠*j*∥*x*_*j*_−*x*_*k*_∥ as the index of *x*_*j*_’s nearest sample, and we regard *Class*_*x*_(*j*) as the label for sample *x*_*j*_. Based on the definition above, *IGP* of group *u* based on dataset *X* is defined as

$$IGP(u,X) = \frac{\#\left\{j|{Class}_{x}\left(j\right) = {Class}_{x}\left(j^{N}\right)=u \right\}} {\#\{j|{Class}_{x}\left(j\right)=u\}}. $$

The value of *IGP* ranges from 0 to 1, and a higher value indicates a better cell distinction between groups.

Because of the heterogeneity of cells, the cells at distinct time stages may still belong to the same cell type, which means that *IGP* tends to underestimate the performance of cell-specific distinction. Consequently, we weaken the requirements of *IGP* and propose a new IGP score by defining the proportion of samples in a group, the nearest neighbors of which are also in the same, or adjacent group (adjacent time stages here). The so-called *IGP2* of the adapted index is formulated as

2 $$\begin{array}{*{20}l} IGP2(u,X) = \frac{\#\left\{j| |{Class}_{x}\left(j\right)-{Class}_{x}\left(j^{N}\right) | \leq 1,{Class}_{x}\left(j\right)=u\right\}}{\#\{j|{Class}_{x}\left(j\right)=u\}}. \end{array} $$

In this study, the tSNE algorithm is implemented by the *Rtsne* function in the R package **Rtsne** with default parameters, and the EE algorithm is performed according to the code downloaded from http://faculty.ucmerced.edu/mcarreira-perpinan/software.html.

## Results

We analyze two time series datasets: Zebrafish [6] and HPE [5] (see “Methods” section for details). To validate the performance of TSEE, we compare it with three other dimensionality reduction algorithms, PCA, tSNE and EE.

### TSEE outperforms other dimensionality reduction methods in visualization and structure preservation

**Fig. 1:**
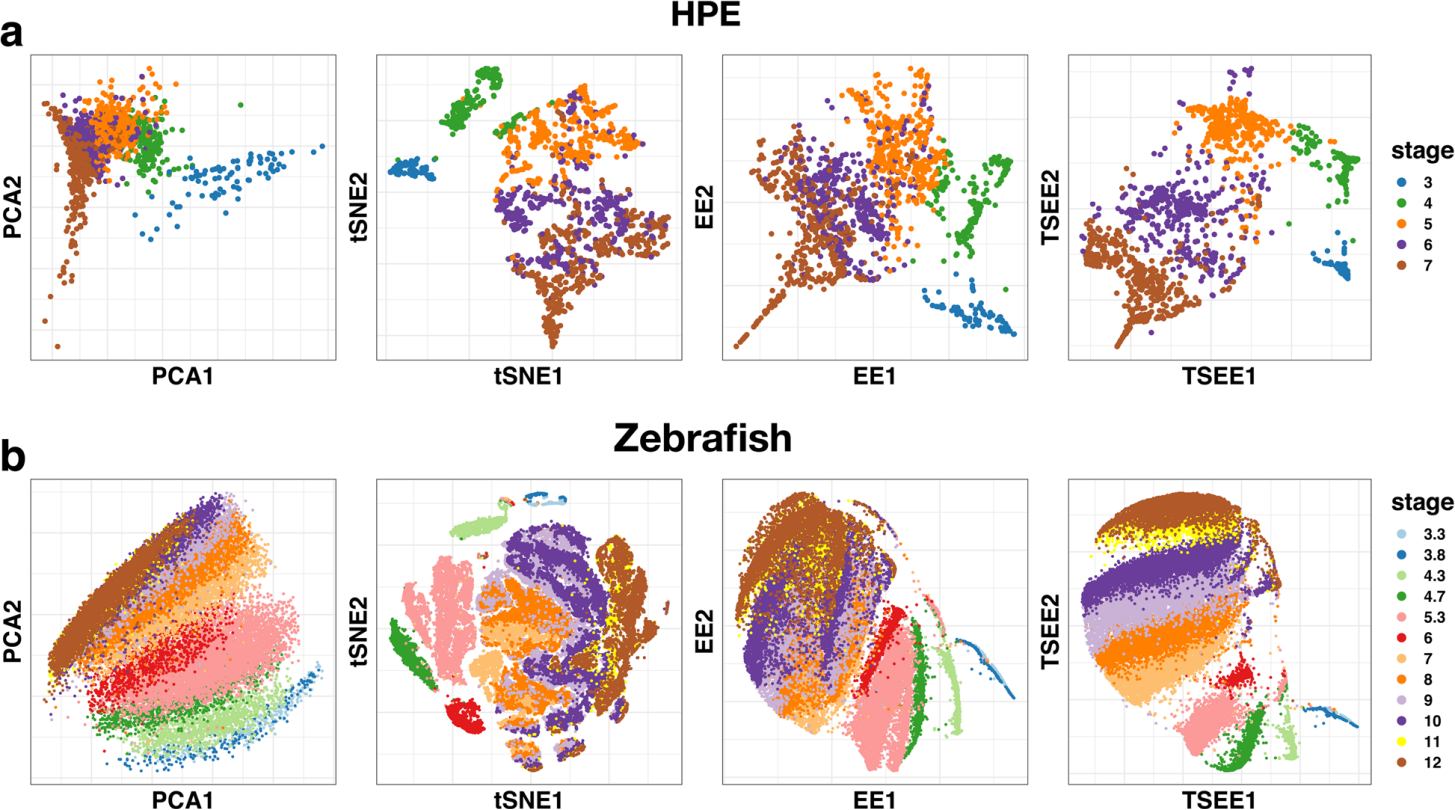
Visualization results by PCA, tSNE, EE and TSEE. The four dimensionality reduction methods are applied to two datasets of time series scRNA-seq: **a** HPE and **b** Zebrafish. Each cell is colored according to the corresponding experimental time stage

We apply the 4 dimensionality reduction algorithms noted above to HPE and Zebrafish datasets and demonstrate the embedding results on the 2-dimensional embedding space to visualize cell development structures. TSEE not only preserves the local and global data structures, but also has higher temporal resolution of cells with different time stages (Fig. 1).

More specifically, in the HPE dataset, PCA combines cells from E4 to E7 tightly, grouping cells into two groups visually, one mainly containing cells at E3 and the other filled with cells from E4 to E7, which would affect downstream analysis, such as clustering. The embedded points with various stages obtained by tSNE and EE are both evenly distributed as a whole, but cells from E5 to E7 heavily overlap, with some E7 cells even falling into the area of E5 cells (Fig. 1a). For TSEE, cells at different time stages are well separated so that only cells at adjacent time stages are mixed in agreement with the heterogeneity of cells (Fig. 1a).

For the Zebrafish dataset, all four methods arrange cells along time with relatively large gaps arising in early developmental stages (Fig. 1b). However, PCA and EE results show that cells from 8 to 12 hpf are highly mixed, almost stacked together, even when two or more time intervals exist among them. Although clearly separating points with different time stages, tSNE shows distorted 2-dimensional temporal structures in that cells from 3.3 hpf to 4.3 hpf are distributed along time course, while cells at 4.7 hpf are separated with the cells before 4.7 hpf by cells at 5.3 hpf (Fig. 1b). In contrast, for TSEE, the cells from 3.3 to 6 hpf are obviously separated. Even though cells from 7 hpf to 12 hpf are not separated as distinctly as the cells before 6 hpf, significant gaps still exist between cells at adjacent time stages, thus showing the best performance on preserving structures along time (Fig. 1b).

**Fig. 2:**
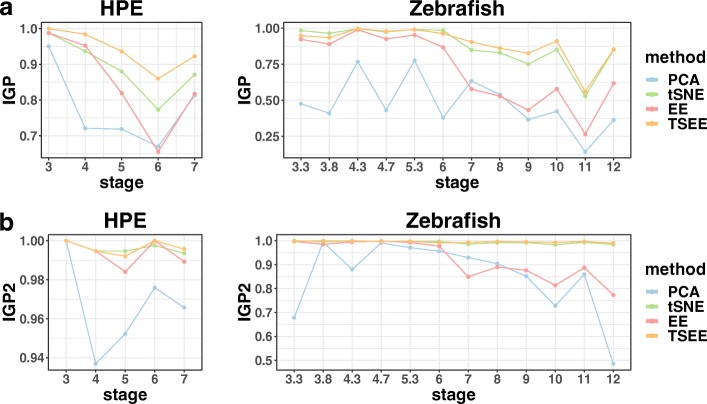
Comparison of local structure preservation performance by *IGP* and *IGP2*. Performances in local structure preservation of PCA, tSNE, EE and TSEE is individually evaluated based on two metrics, **a***IGP* and **b***IGP2*, on the HPE and Zebrafish datasets

To quantitatively evaluate the performance of TSEE, we apply *IGP* and *IGP2* scores to measure the distinction of cells, regarding time stages as standard of cell groups. TSEE always has highest averaged values of *IGP* and *IGP2* (see Tables 1 and 2), and its performance is similar to that of tSNE based on the two scores across all time stages in HPE and Zebrafish (Fig. 2), indicating that TSEE preserves the local structures and differentiates cells at various time stages better than the three other methods.

**Table 1:** Evaluation of local structure preservation based on averaged values of *IGP*

	PCA	tSNE	EE	TSEE
HPE	0.7747	0.8900	0.8466	0.9407
Zebrafish	0.4757	0.8799	0.7127	0.8943

**Table 2:** Evaluation of local structure preservation based on averaged values of *IGP2*

	PCA	tSNE	EE	TSEE
HPE	0.9661	0.9961	0.9936	0.9965
Zebrafish	0.8525	0.9930	0.9196	0.9954

**Fig. 3:**
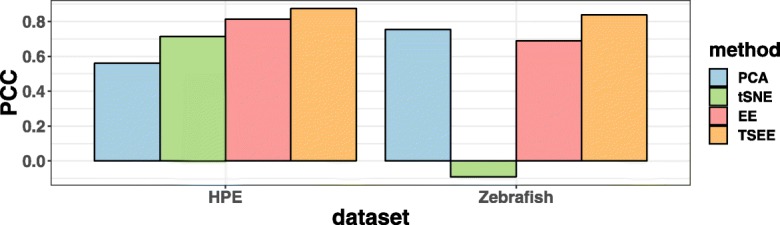
Quantitative comparison of global structure preservation. Performance in global structure preservation of PCA, tSNE, EE and TSEE is evaluated based on PCC metric of pseudotime calculated by DensityPath and experimental time for the HPE and Zebrafish datasets

To evaluate the preservation of global and temporal structures by the four methods, we measure performance quantitatively by applying DensityPath [14], which has high accuracy in the reconstruction of cell development trajectory, as well as pseudotime calculation, for both datasets. Since our purpose is to investigate the performance of the four dimensionality reductions, we only apply DensityPath to dimensionality reduced data of 2-dimensional space by the four methods to reconstruct cell state-transition path and calculate pseudotime. After setting the root as 1472-th and 1-st cell in the HPE and Zebrafish datasets, respectively, the accuracy of calculated pseudotime is measured by PCC between the calculated pseudotime and experimental time of the cells. A larger value of PCC indicates more consistency in global structure preservation along time. We find that TSEE preserves temporal structure information best, while the PCC value of tSNE on the Zebrafish dataset is negative, indicating that the embedding of cells by tSNE distorts whole structures of data along time (Fig. 3).

**Fig. 4:**
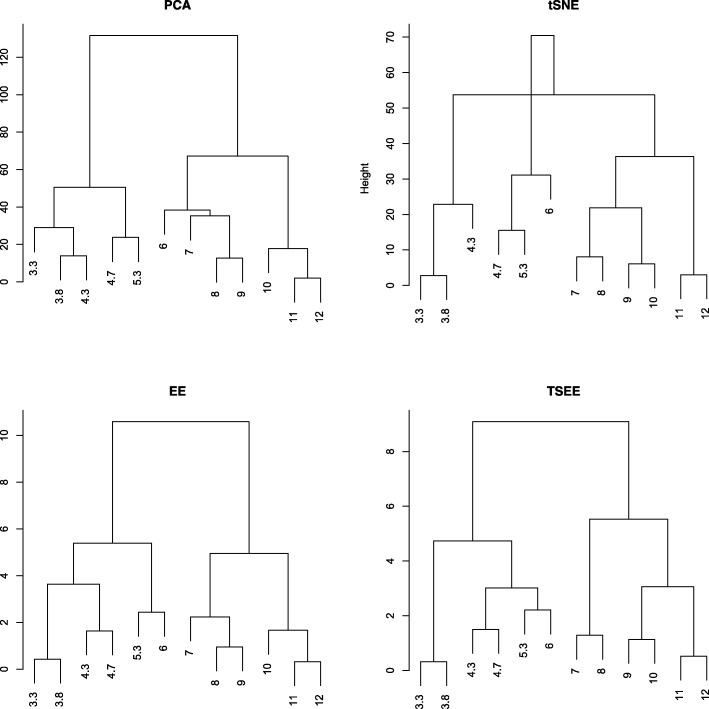
Hierarchical cluster tree of centroids at various time stage, as obtained by PCA, tSNE, EE and TSEE. The centroids of samples with different time stages are submitted to hierarchical clustering to validate the preservation of time order for the Zebrafish dataset

The hierarchical clustering algorithm is also applied to measure the preservation of distance of time stage. The complete linkage method is adopted here based on centroids of samples at each time stages, where the calculated distance of the centroids is based on 2-dimensional Euclidean distances of the four dimensionality reduction methods separately. Although the adjacent time stages are always close to each other in hierarchical clustering trees based on the results of all four methods (Fig. 4), the clustering tree constructed on the basis of TSEE results shows the best consistency between the distance of centroids and the time order. Specifically, the lengths of leaf nodes (3.3, 3.8, 4.3, 4.7, 5.3, and 6) to the root of the clustering tree decrease with increasing time stage from 3.3 to 6 hpf, and then the lengths of leaf nodes (7, 8, 9, 10, 11, and 12) to the root of clustering tree increase with the increasing time stage from 7 to 12 hpf (Fig. 4). As an indication of cell development processes, the clustering tree result by TSEE indicates that the centroids of cell populations propagate along one direction from 3.3 to 6 hpf and then propagate along another direction from 7 to 12 hpf, which can be further supported by the large gap between 6 and 7 hpf on the 2-dimensional space by tSNE, EE, and TSEE (Fig. 1b).

### TSEE reveals subtle dynamic patterns of gene expression during zebrafish embryogenesis on 2-dimensional space

The large-scale Zebrafish dataset with closely spaced stages allows us to further explore dynamic patterns of gene expression during zebrafish embryogenesis.

**Fig. 5:**
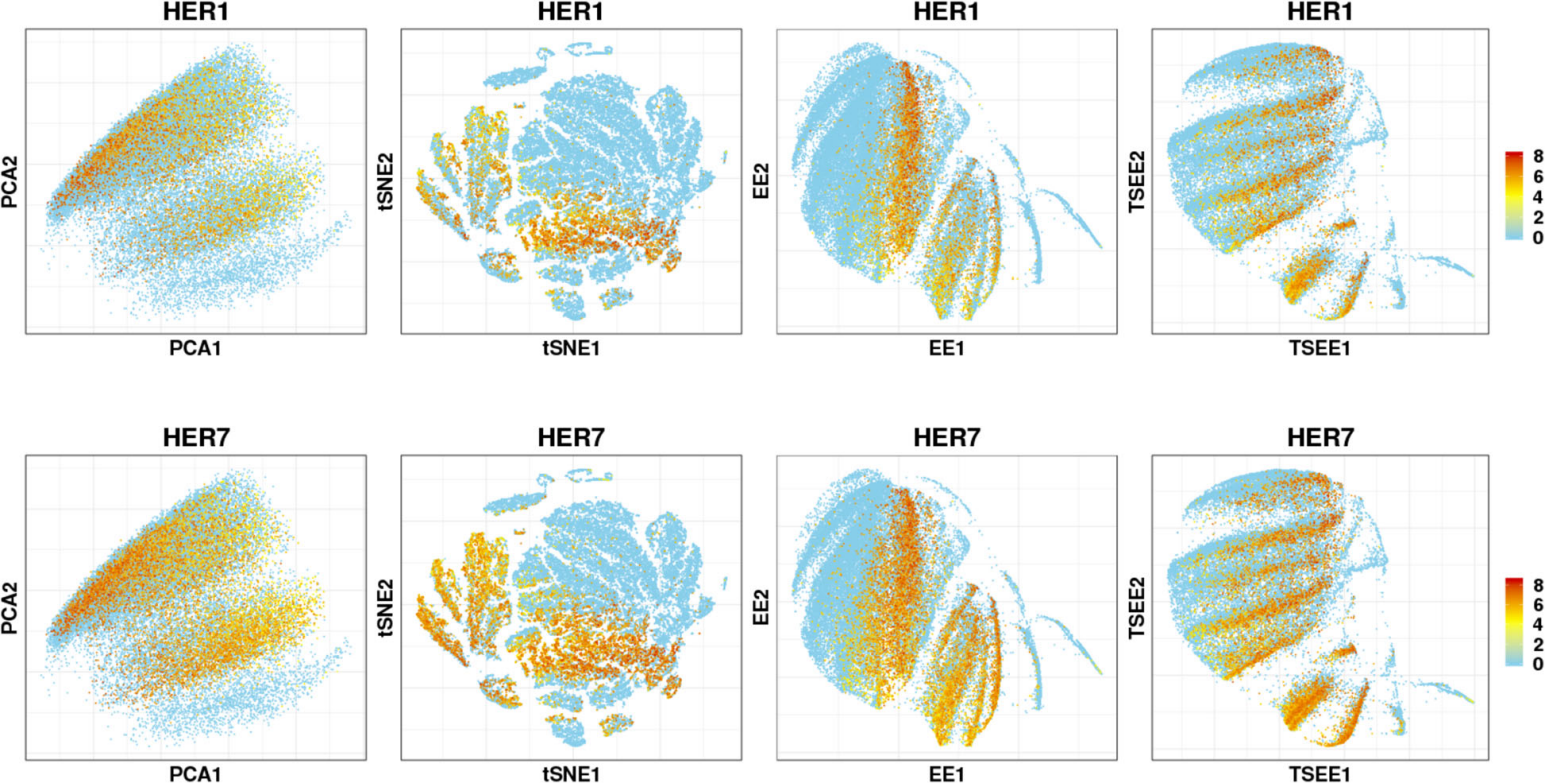
Distribution of gene expression of *HER1* and *HER7* in 2-dimensional space. The distribution of *HER1* and *HER7* gene expression in 2-dimensional embedding obtained by PCA, tSNE, EE and TSEE, revealing the oscillatory expression pattern along the development of time course during zebrafish embryogenesis

The power of TSEE to allow visualization of structures that show dynamic patterns of gene expression can first be illustrated through the expression of marker genes *HER1* and *HER7* on the 2-dimensional space by PCA, tSNE, EE and TSEE in Zebrafish data (Fig. 5). *HER1* and *HER7*, for which the existence of oscillation expression patterns by negative feedback has been reported in presomitic mesoderm of zebrafish [23], clearly show oscillatory expression patterns along time stages on the 2-dimensional space of TSEE, indicating that TSEE successfully reveals the underlying dynamic patterns of gene expression. On the other hand, PCA only shows two wide strips of highly expressed genes on the whole plot, and tSNE gathers cells with highly expressed genes together without any oscillation pattern whatsoever. Meanwhile EE just shows a narrow region with oscillation expression pattern in early developmental stages, and the cells with high-level expression are stacked in the latter developmental stages, resulting in low temporal resolution. For TSEE, however, points on the 2-dimensional plane clearly demonstrate the oscillation patterns of gene expression with high resolution, validating the effectiveness of TSEE in preserving the intrinsic structures of data with high resolution, while, at the same time, allowing visualization of the underlying dynamic structures of genes.

**Fig. 6:**
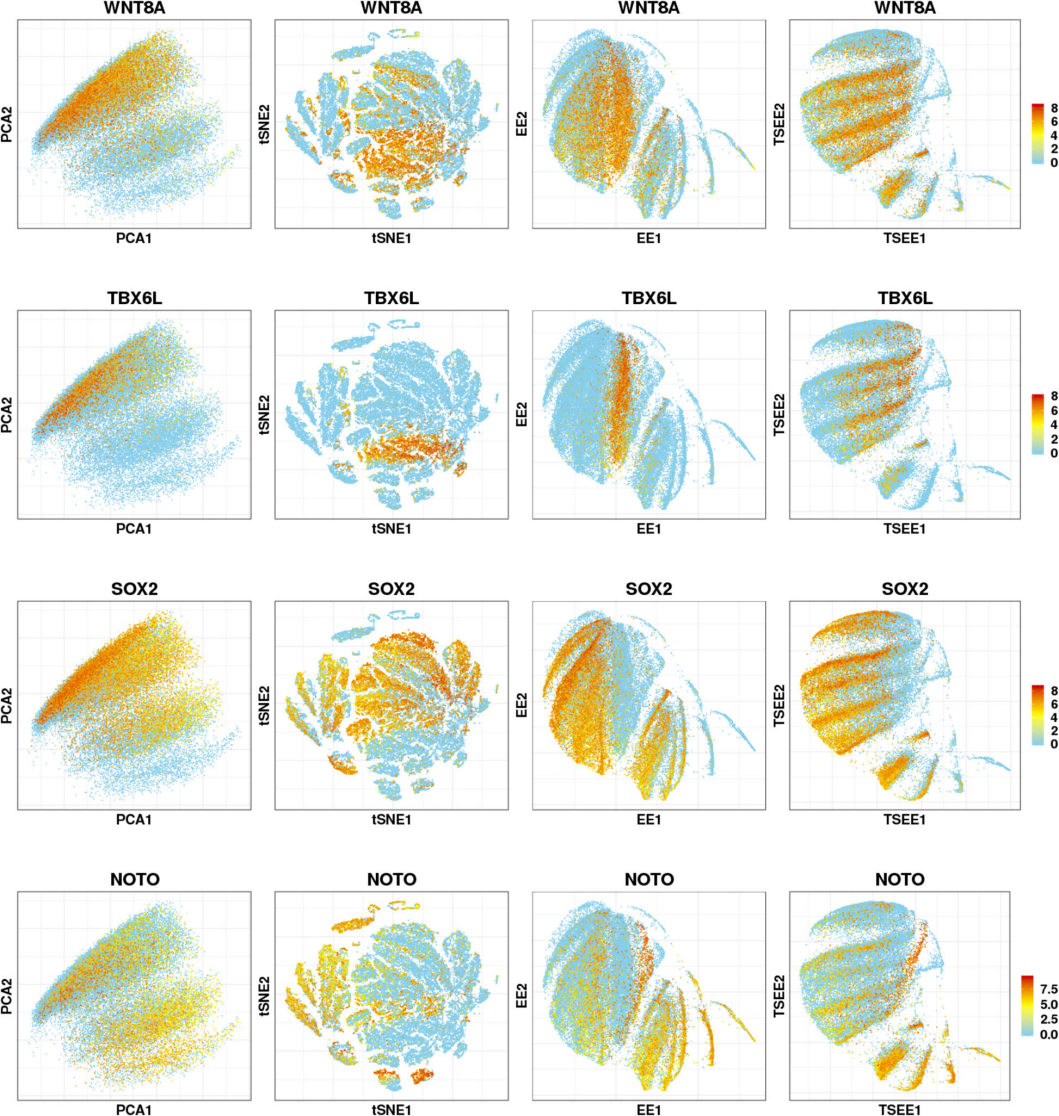
Distribution of expression of four genes in 2-dimensional space. Distribution of expression of four genes in 2-dimensional embedding obtained by PCA, tSNE, EE and TSEE. Dynamic gene expression patterns are revealed by TSEE

We also explore the expression patterns of genes related to embryogenesis from the list provided by [6] on the 2-dimensional spaces by the four methods to examine the developmental patterns. Here, visualization by TSEE can also show clearer oscillation waves of gene expressions along time compared to the other methods (see Fig. 6 for four examples). PCA tends to distribute the cells with high-level gene expression uniformly, or accumulate them in the region of late developmental stages. The tSNE methods tends to gather together the cells with high gene expression, while small gaps occur among them, but this only shows meaningful clusters of cells without any evidence of patterns of gene expression. EE displays the products resulting from mixtures of cells at various time stages as the region of high-level gene expression, where genes are continuously expressed. The three methods place together the regions where the specific genes are highly expressed, failing to uncover the dynamic structures even if some special gene expression patterns exist among all cells. In TSEE, different patterns of gene expression can be revealed. As examples, *WNT8A*, an essential transcript for zebrafish axis development [24], displays an oscillatory gene expression pattern throughout all samples. *TBX6L*, which contributes to posterior paraxial mesoderm formation during zebrafish embryogenesis [25], reflects a periodically expressed pattern in the right region. *SOX2*, identified as an essential transcription factor to maintain self-renewal or pluripotency [26], reflects oscillation, as well, in the left region in the whole stages. Finally, *NOTO* displays a periodic gene expression pattern entirely, but fades away gradually along time. More genes with an oscillating pattern if expression can be found at https://github.com/ShaokunAn/TSEE.

Oscillating patterns of gene expression revealed by TSEE may be linked to the genome-scale oscillations in DNA methylation during exit from pluripotency [27]. Therefore, our TSEE results provide new insights and perspectives for subsequent analysis of dynamic transitions and regulation mechanisms of key genes.

### Parameter choices and robustness analysis of TSEE

Two tuning parameters, *λ* and *β*, are found in the pseudo potential energy function *E* of TSEE (Eq. 1). The parameter choices of *λ* and *β* are critical to the embedding results by TSEE. In the implementation of TSEE, we set the default values of both *λ* and *β* equal to 10.

**Fig. 7:**
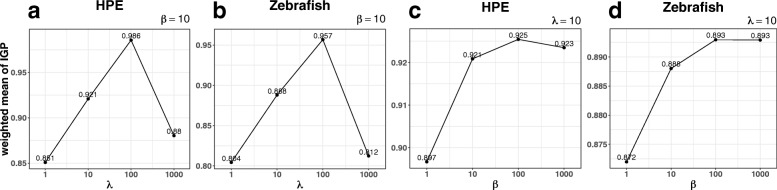
Weighted mean of *IGP* under different parameter values. The y-axis is the weighted mean of *IGP* where the weights are the proportions of the number of cells at each time stage. The x-axis represents parameter choices (in logarithmic scale). (**a**) results of different *λ* for HPE dataset, (**b**) results of different *λ* for Zebrafish dataset, (**c**) results of different *β* for HPE dataset, (**d**) results of different *β* for Zebrafish dataset

To test the robustness of the parameter choices, we first choose the values of each parameter separately in a wide range from 1 to 1000, while keeping the other parameter fixed at default, and calculate weighted mean of *IGP* scores for both HPE and Zebrafish datasets (Fig. 7). The weighted mean of *IGP* is the sum of *IGPs* at each time stage, weighting by the proportion of samples (cells) at each time stage.

**Fig. 8:**
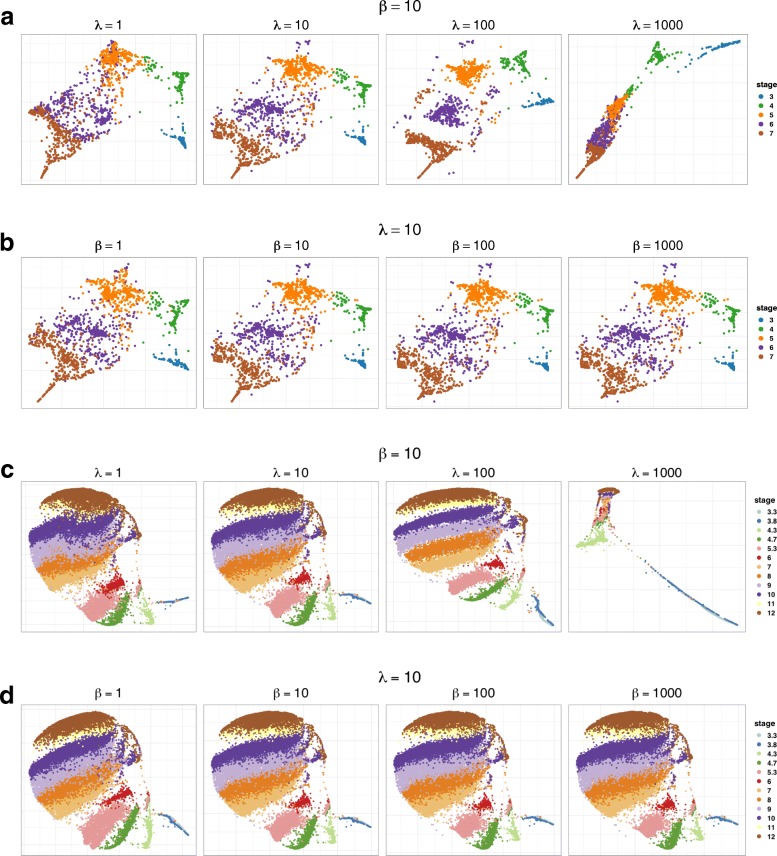
The 2-dimensional embeddings of TSEE under different parameter settings. The visualization results of TSEE by varying one parameter, while the other is fixed at the default value of 10, are displayed on **a**, **b** for the HPE dataset and on **c**, **d** for the Zebrafish dataset

When fixing *β*=10, TSEE achieves the highest weighted mean of *IGP* at *λ*=100 in both datasets (Fig. 7a,b). However, the large *λ*(≥100) tends to separate samples into clusters on the 2-dimensional space of TSEE, breaking the structure into discontinuities (Fig. 8a,c), which is inappropriate for continuous embryonic development. Therefore, *λ*=10 best balances the preservation of local and global structures.

When fixing *λ*=10, the weighted mean of *IGP* increases sharply when increasing *β* from 1 to 10 and tends to be saturated after *β*≥100 (Fig. 7c,d). Although the global structures of cells on the 2-dimensional space of TSEE are stable by varying *β* from 1 to 1000, TSEE may sacrifice some local structures with large *β*(≥100) since the repulsive terms are determined by the difference between time stages, while the dissimilarities based on gene expression are seldom considered. For example, the two corner sections at stage E7 and the one at stage E4 become less distinguishable as *β* grows in HPE data. Therefore, *β*=10 is sufficient for the incorporation of temporal information data and trades off the weights between gene expression and time stage.

**Fig. 9:**
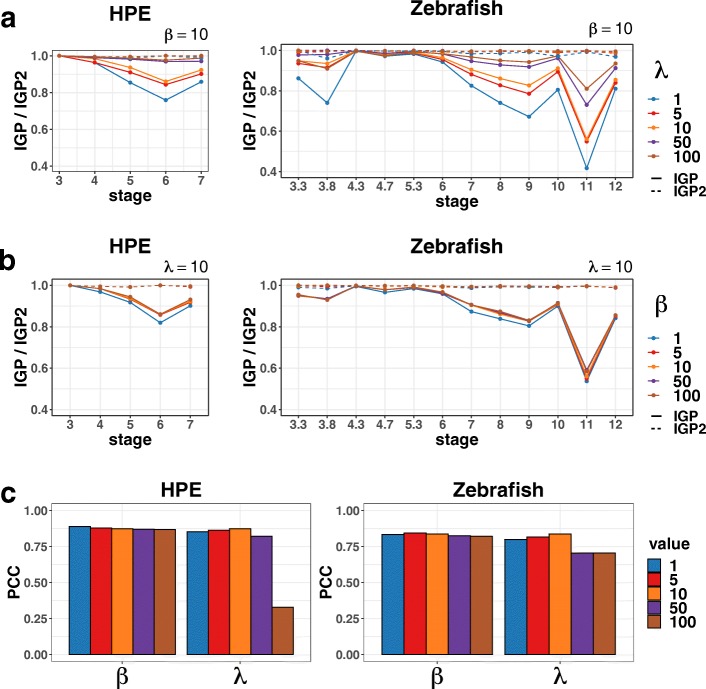
Robustness analysis of parameters on two datasets. Robustness analysis is performed for *λ* and *β* on the two datasets. To evaluate preservation of local structure under each set of parameters, *IGP* and *IGP2* are used. **a** When *β* is set as default 10, *IGP* and *IGP2* display the effect by the change of *λ*. **b** When *λ* is set as default 10, *IGP* and *IGP2* display the effect by the change of *β*. **c** PCC is used to assess the preservation of global and temporal structures

To determine the parameters more precisely, as well as analyze the robustness of TSEE, we further study the performance of TSEE when the parameters are tested in detail in the region from 1 to 100. The three metrics *IGP*, *IGP2* and PCC are employed here. Based on *IGP* and *IGP2*, TSEE has similar performance for various *β* and *λ* from 1 to 50 in the HPE and Zebrafish datasets, indicating that TSEE is quite robust to the change of parameters on preserving local structures. We calculate PCC for HPE and Zebrafish data to analyze the robustness to parameter choice, as well. Figure 9 demonstrates that TSEE is quite robust for the choices of *β*, as well as choices *λ*, when varying them separately in the range from 1 to 50.

Based on all the results above, we set the optimal default values of both *β* and *λ* to be equal to 10.

## Discussion

In summary, we propose a novel visualization method, TSEE, for time series scRNA-seq data. To the best of our knowledge, in the single-cell community, TSEE is the first visualization method that considers additional temporal information.

**Fig. 10:**
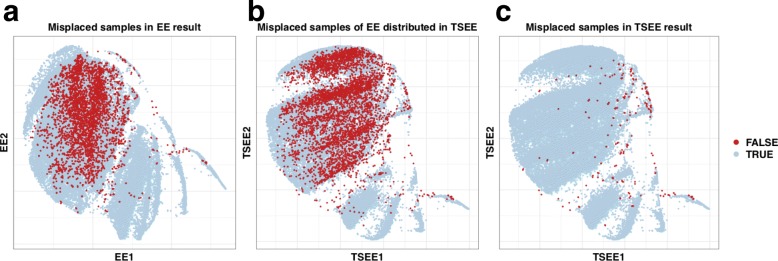
Distribution of misplaced samples based on 2-dimensional embeddings. When one cell’s nearest neighboring point is at least two time intervals away from it, the cell is considered to be misplaced with high probability and is colored in red in above figures. **a** shows the distribution of misplaced cells by EE on latent space of EE, **b** displays the distribution of misplaced cells by EE on latent space of TSEE, and **c** displays the distribution of misplaced cells by TSEE on latent space of TSEE

TSEE enhances resolution of the map by correcting for unknown noise variation. The repulsive force based on time intervals of samples enables TSEE to align cells along time, preserving temporal structures, while the intrinsic structures remain well preserved owing to the incorporation of the attractive term and the repulsive term based on their distance in gene expression space. Figure 10 displays the correction of cells by TSEE. The samples colored in red are those for which the nearest neighboring points are at least two time intervals apart, indicating with high probability that they are misplaced in the 2-dimensional plane. The misplaced points by EE are distributed uniformly (Fig. 10a), but when displayed on the 2-dimensional space obtained by TSEE (Fig. 10b), they are aligned into their respective time stages, indicating that TSEE corrects the misplacement of samples by EE. Moreover, the misplaced cells by TSEE are generally located in the interface of two time stages (Fig. 10c), which makes sense since cells are typically heterogeneous, especially at late developmental stages. Because of the correction of cells along time, gene expression patterns can be revealed, such as the oscillation process of *HER1* and *HER7* in Zebrafish data, as demonstrated in the “Results” section. The increasing number of genes with oscillatory expression pattern discovered is further supported by the genome-scale oscillations in DNA methylation[27], and the uncovered subtle dynamic structures of time series scRNA-seq data, as, for example, through the use of TSEE, can be utilized for further analysis of gene expression.

The visualization tool of the forced-directed layout [15] utilizes the concept of attractive force and repulsive force, as well, to visualize network structures in low-dimensional space. The data tend to be collapsed along branches, covering cell-to-cell heterogeneity, as well as latent gene expression pattern, and the results in low-dimensional space generally need to be adjusted manually. For example, the marker *WNT8A* demonstrates an oscillating expression pattern in the TSEE visualization result, but the development tree result in the Supplementary File of [6], which was obtained by a hand-tuned force-direct layout merely shows a growing tendency along time, and the heterogeneity of cells is hidden because cells collapse along branches.

**Fig. 11:**
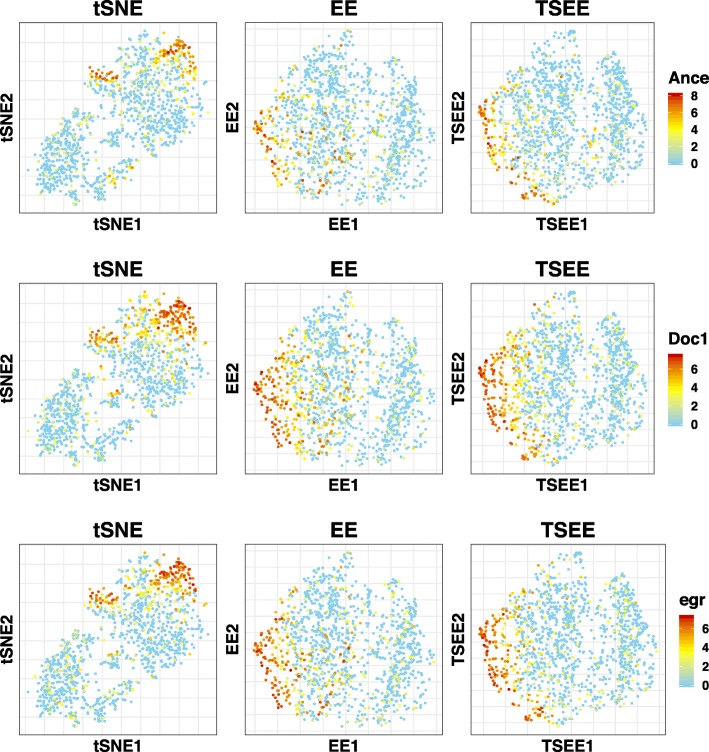
Distribution of genes expressed in *Drosophila* embryo cells. The expression of *Ance*, *Doc1* and *egr* are displayed in 2-dimensional space, as obtained by tSNE, EE and modified spatial TSEE

The computational framework of TSEE can also be potentially extended to incorporate other sources of information (e.g., spatial information) of scRNA-seq data. We utilize the scRNA-seq data of embryo cells from [21] to demonstrate the extended application based on spatial information of cells. A set of 84 marker genes are considered to uniquely classify almost every position within the embryo [21]. Therefore, we employ the information from the 84 marker genes to quantify the spatial distance of single cells, defining as 1−PCC(*x*_*i*_,*x*_*j*_) between samples *i* and *j*, where *x*_*i*_ and *x*_*j*_ are the expression vectors of the 84 marker genes. Figure 11 displays the expression of three markers of dorsal ectoderm in 2-dimensional space, as obtained by tSNE, EE, and TSEE, respectively. Compared to the results of EE and tSNE, the highly expressed genes tend to locate along the boundary of the 2-dimensional space by the modified spatial TSEE, showing better consistency with the spatial patterns of the genes in the embryo. The accurate reconstruction of spatial location of single cells will be challenging, and this will be an interesting topic for our future study.

## Conclusions

In this study, we propose an efficient algorithm, TSEE, for the visualization of time series scRNA-seq data. TSEE is an extension of EE by introducing an additional repulsive force term when pairs of data points are collected at distinct time stages, thereby balancing the effects from disparities between samples in high-dimensional gene expression space.

To incorporate the temporal information of data, TSEE adds additional terms of the time intervals between samples into the repulsive terms to balance the effects from the disparities between samples in high-dimensional gene expression space. In this way, TSEE dilutes the distortions of the assorted sources of variations of the data across time stages and achieves temporal resolution enhancement by preserving temporal order and structure. In addition, TSEE uncovers the subtle dynamic structures of gene expression patterns, as exemplified by oscillating waves in our results, facilitating further downstream dynamic modeling and analysis of gene expression processes. The computational framework of TSEE is generalizable for the incorporation of other sources of information.

## Abbreviations

EE: Elastic embedding
HPE: Human preimplantation embryo
IGP: In-group proportion
IGP2: A modification of in-group proportion
PCA: Principal component analysis
PCC: Pearson correlation coefficient
scRNA-seq: Single-cell RNA sequencing
TSEE: Time series elastic embedding
tSNE: t-distributed stochastic neighbor embedding
